# Vertebral column resection for severe kyphosis

**DOI:** 10.3171/2020.1.FocusVid.19752

**Published:** 2020-01-01

**Authors:** Munish C. Gupta

**Affiliations:** Department of Orthopedic Surgery, Washington University in St. Louis School of Medicine, St. Louis, Missouri

**Keywords:** congenital kyphosis, vertebral column resection, severe kyphosis, correction of kyphotic deformity, video

## Abstract

Vertebral column resection is an excellent tool for the correction of sharp angular deformities. Preoperative planning is crucial, and a 3D model is helpful. The spinal column is stabilized before any resection is performed. The dorsal fusion mass holds the dura and spinal cord suspended with the adhesions while the anterior decompression is being performed. The correction is performed by shortening with compression, rod exchange, in situ bending, cantilever, and compression. The anterior column support is important. Multiple rods prevent early rod failure. The cord is covered with bone graft to prevent mechanical compression from muscle or a hematoma.

The video can be found here: https://youtu.be/FlBE5SFa2Gw.

## Transcript

### 0:20 Introduction and approach

Vertebral column resection for severe kyphosis.

Thirty-three-year-old female who has congenital thoracolumbar kyphosis. The patient had a posterior spinal fusion with instrumentation when she was 11 years old. The instrumentation got loose and was prominent. She had removal of instrumentation, and ever since she has had increasing kyphosis. She has pain with sitting and wakes up at night with back pain. She has right buttock pain and posterior thigh/leg pain radiating down the leg. The motor and sensory exam is normal. Pain on palpation in the thoracic spine above and below the gibbus. MRI scan of the thoracic spine showed a small syrinx.

These are plain radiographs of the patient showing the severe thoracolumbar kyphosis. The 3D model not only demonstrates the different anatomy that is seen from right versus left, but posteriorly, the fusion mass is well visualized and the small defect in the fusion mass is seen, which can be problematic if one is not expecting that in the approach. This is a clinical photograph of the patient showing the previous incision and the thoracolumbar kyphosis. She has compensated with hyperlordosis for the thoracolumbar kyphosis as well as lack of thoracic kyphosis.

The approach is first made; all the soft tissues are stripped and the facetectomies are performed with an osteotome. The burr is then used to define the entry point for the pedicle screws. A pedicle probe is used to cannulate the pedicle. A ball-tip probe is used to make sure there’s no violations of the pedicle wall, and then the appropriate size and length of screw is placed. This is done in the thoracic spine and you can see now that all the screws are in position.

### 1:22 Smith-Petersen osteotomies

In the lumbar spine, the Smith-Petersen osteotomy is performed by removing some of the fusion mass that is overgrown with an osteotome. This is done on both sides and then the canal is encountered. A rongeur is used to remove the excess bone. A Kerrison rongeur is then used to remove the ligamentum flavum as well as the facet capsule and the facet joint partially to go through the foramen to perform a release of the posterior elements. There is plenty of mobility achieved at the osteotomy site.

The next step is placement of the pedicle screws in the lumbar spine by decorticating the transverse process for a fusion and also to make the entry point for the lumbar screws. The lumbar pedicles are then cannulated with a pedicle probe, checked with a ball-tip probe, and then the appropriate size and length of screw is placed up and down the lumbar spine.

After all the screws are placed in the thoracic and lumbar spine, additional Smith-Petersen osteotomies are performed proximally over the fusion mass. First, the aggressive burr is used. Then the diamond-tip burr is used until the ligamentum flavum. The ligamentum flavum is then removed and the Kerrison rongeur is taken through the foramen.

### 2:34 Rib dissection, disconnecting the dorsal lamina, vertebral body dissection

The ribs are dissected. As you can see, the ribs are quite different in appearance. The ribs are then cut leaving a small portion of the rib attached to the rib head. The foramen is then found and then the canal is entered laterally to medially and a burr is used to thin down the lamina on the lateral surface where the canal is entered. After this is done, the rongeur is used to debulk the bone. More of the bone is thinned down with a burr, and a Kerrison rongeur is used to disconnect the dorsal lamina from the vertebral bodies. The Woodson is used to find where the pedicles are and where the canal is. In this way, the pedicles are identified and the Kerrison rongeur is used to remove the pedicles. The dorsal lamina is completely disconnected from the vertebral bodies that are going to be resected.

The rib heads are then isolated. The pleura is bluntly dissected off the rib heads and the rib heads are then removed sequentially. Nerve roots are identified and dissected and then sacrificed after tying off the proximal and distal ends of the nerve roots. You can see now that the rib heads are removed from both sides. Now the lateral portion of the spine is approached. Hemostasis is achieved with bipolar cautery and dissection is carried out of the lateral portion of the spine.

Blunt dissection can be used to expose the anterior portion of the spine. All of the soft tissue is dissected away from the anterior portion of the spine. Sponges are used to protect the soft tissue from the anterior portion of the spine. Bipolar and electrocautery is used to maintain hemostasis. The Woodson elevator is now displaying how the canal has been entered and the dorsal lamina is completely free from both sides of the vertebral bodies.

The vertebral body resection can be started. Before resecting the vertebral bodies, a temporary rod is placed to stabilize the spinal column. So, no sudden movement can produce a neurologic injury. Ribbon retractors and gauze sponges can be used anterior to the spine to protect the soft tissues. The canal is now exposed on the other side in a similar manner. Disconnecting the dorsal lamina removing the pedicles, exposing the nerve roots and maintaining the dorsal lamina that is still connected to the spinal cord with some adhesions. Additional nerve roots can be sacrificed, but before sacrificing the nerve roots they need to be tied on both sides. You can see the ribbon retractor that is all the way across the anterior portion of the spine.

### 5:22 Vertebral body removal

Now the vertebral bodies are decancellated with a burr and various sizes of curettes. In this portion of the video, you can see the posterior wall is still intact. The vertebral bodies have been removed. Now the disc spaces are identified and the cartilaginous endplate is removed with a Cobb elevator, rongeur, and curettes. This provides a very clean, bony surface superiorly and inferiorly where the anterior spinal fusion is going to take place.

### 6:04 Removal of the posterior wall

Now the posterior wall is intact still and the lateral portion of the posterior wall is removed with large pituitary rongeur. Now you can see that the posterior wall is being dissected away from the dura, so there’s no adhesion between the spinal cord and the posterior wall. A down-going curette is used to push down the posterior wall away from the spinal cord. Any bony fragments or soft tissues is removed. Now you can see the pulsations of the dura with complete resection of all the bone anterior to the spinal cord. There is a significant angulation that can be appreciated where the cage is going to go, and our goal would be to take those endplates and make them parallel before the cage is placed anteriorly. Now the second rod is replaced again.

### 7:02 Removing the dorsal lamina and decompressing the spinal cord

After all this is done, the dorsal lamina is then freed completely and removed from the posterior part of the dura, taking away all the adhesions. This actually is a very safe way to hold the spinal cord while anterior decompression is being performed. The adhesions between the dorsal lamina and the spinal cord and dura hold the dura and prevent it from falling down so you’re not constantly bumping into it when you’re removing the posture wall. Now the spinal cord is free. You can see pulsations. You can see the dura is posteriorly thoroughly decompressed and anteriorly is thoroughly decompressed, as shown in this picture, and the angulation of the virtual bodies are quite apparent. Any soft tissue or bony elements remaining are checked and removed.

### 7:56 Correction of the kyphosis

Now compression is applied to the rods sequentially to shorten the deformity and relax the spinal cord in this manner. Sequential rods are exchanged a couple of times by taking out the rod, under bending it, and then placing it on one side and then replacing it on the other side. To correct the kyphosis, in situ bending is then performed. This maneuver eliminates the kyphosis but also angulates the bodies from being at a sharp angle to more parallel. Additional compression is then applied to these temporary rods to create more correction of the kyphosis. Cantilever, compression, and also in situ bending is used to correct the kyphosis. Now the final rods are placed, but before doing that one rod is left in place. Additional stability is applied with a distractor holding the two vertebral bodies distracted. The rod on the other side is then cut to size, contoured and anchored proximally, and then with cantilever maneuver it’s dropped down into the lower part of the construct into the lumbar spine. Additional compression is also applied after placing the other rod. This really shows the final correction of the kyphosis.

### 9:23 Anterior cage placement

Now anterior vertebral bodies are more parallel and a cage is sized and placed from endplate to endplate. This photograph shows the placement of the cage and the correction of the kyphosis, the shortening posteriorly and the lengthening anteriorly of the spinal column. This is a photograph of the image intensifier showing the placement of the cage between the two vertebral bodies that are now parallel that were so severely angled at the time of resection. The kyphosis is also corrected. This is a photograph showing the final construct with just two rods, and additional rod is placed across the three-column osteotomy to make it more mechanically sound and prevent early rod failure.

### 10:00 Placement of the bone to protect the cord and fusion, radiographic and clinical result

The dorsal lamina that was resected from the fusion over the kyphosis is fashioned, shortened, and placed over the dura that’s exposed and tied down with suture to hold it in. Some grooves are made into the lamina to prevent the sutures from slipping. This provides protection of the exposed spinal cord from mechanical compression as well as hematoma formation and compression. At this point, the spine is decorticated, bone graft is placed over the lamina, and bone graft is also placed anteriorly on the sides of the cage. Off-label use of BMP-2 is shown here to provide a solid fusion. This is the final photograph showing the construct multiple rods, dorsal lamina protecting the open spinal cord area that provides also an area of fusion, and then all the bone and BMP placed proximally and distally. What is not shown is the anterior bone graft that is placed adjacent to the cage on the sides. This radiograph shows the correction of the thoracolumbar kyphosis. There is a small coronal deformity that’s residual in the lumbar spine, but there’s a significant improvement of the thoracolumbar kyphosis. This photograph shows the clinical improvement of the thoracolumbar kyphosis in the patient.

### 11:11 Summary

Vertebral column resection is an excellent tool for posterior correction of sharp angular deformities even in a previously fused setting since we can control the anterior and posterior part of the vertebral column simultaneously. The preoperative planning is crucial, and one should consider a 3D model if possible because it aids in planning as well as execution of the surgical plan in the safe and effective manner. All the fixation points should be placed before any resection is performed. When the resection is performed, stabilization with the rods is important so there’s no sudden change in the vertebral column alignment. Leaving the dorsal fusion mass holds the dura and spinal cord suspended with the adhesions while the anterior decompression is being performed. The correction is then performed by shortening with compression, rod exchange with undercontouring, in situ bending, cantilever, and compression. The anterior column’s support is extremely important. Multiple rods prevent early rod failure in the area where it has the least bony contact and the greatest resection of the bone. The posterior protection of the cord is also important to prevent mechanical compression from muscle or a hematoma.
